# Tyro3 Targeting as a Radiosensitizing Strategy in Bladder Cancer through Cell Cycle Dysregulation

**DOI:** 10.3390/ijms23158671

**Published:** 2022-08-04

**Authors:** Linda Silina, Florent Dufour, Audrey Rapinat, Cécile Reyes, David Gentien, Fatlinda Maksut, François Radvanyi, Pierre Verrelle, Isabelle Bernard-Pierrot, Frédérique Mégnin-Chanet

**Affiliations:** 1 Institut Curie, CNRS, UMR144, Equipe Labellisée Ligue Contre le Cancer, PSL Research University, 75005 Paris, France; 2 INSERM U 1196/CNRS UMR 9187, Paris-Saclay Research University, 91405 Orsay, France; 3 Institut Curie, Bat. 112, Rue H. Becquerel, 91405 Orsay, France; 4 Genomics Platform, Translational Research Department, Research Center, Institut Curie, Paris Sciences et Lettres (PSL) Research University, 75005 Paris, France; 5 Institut Curie-Hospital, Radiation Oncology Department, 75005 Paris, France; 6 Department of Radiation Oncology, Faculty of Medicine, Clermont Auvergne University, 63000 Clermont-Ferrand, France

**Keywords:** bladder cancer, TYRO3, radiosensitivity, TAM receptors, receptor tyrosine kinase, NanoString

## Abstract

Bladder cancer is a common cancer; it is the tenth most common cancer in the world. Around one fourth of all diagnosed patients have muscle-invasive bladder cancer (MIBC), characterized by advanced tumors and which remains a lethal disease. The standard treatment for MIBC is the bladder removal by surgery. However, bladder-preserving alternatives are emerging by combining chemotherapy, radiotherapy and minimal surgery, aiming to increase the patient’s quality of life. The aim of the study was to improve these treatments by investigating a novel approach where in addition to radiotherapy, a receptor, TYRO3, a member of TAM receptor tyrosine kinase family known to be highly expressed on the bladder cancer cells and involved in the control of cell survival is targeted. For this, we evaluated the influence of TYRO3 expression levels on a colony or cell survival assays, DNA damage, γH2AX foci formation, gene expression profiling and cell cycle regulation, after radiation on different bladder cell models. We found that TYRO3 expression impacts the radiation response via the cell cycle dysregulation with noeffets on the DNA repair. Therefore, targeting TYRO3 is a promising sensitization marker that could be clinically employed in future treatments.

## 1. Introduction

Bladder cancer (BCa) is the tenth most common cancer in the world and the fourth most common cancer in men in Europe. It is four times more likely to occur in males and is more frequent in industrialized countries [1]. It is also the most expensive cancer to treat per patient considering the numerous relapses and life-long regular surveillance needed for non-muscle-invasive bladder cancer (NMIBC) [2]. 25% of all diagnosed BCa are muscle-invasive bladder cancer (MIBC), which have very poor outcomes, with an overall 5 year survival of 50–60% for patients with localized disease, and less than 10% for patients in presence of metastasis. In locally advanced or metastatic MIBC, immunotherapy or anti-FGFR targeted therapy for tumors presenting FGFR2 or FGFR3 genetic alterations showed benefits [3,4]. Further, for localized MIBC the standard treatment is cystectomy with adjuvant or neoadjuvant chemotherapy, but is often associated with morbidity and a poor quality of life [5,6].

Radiotherapy coupled with chemotherapy and tumor transurethral resection (bladder-sparing trimodality therapy) has emerged as a radiation-based bladder sparing treatment for localized disease, showing comparable survival outcomes with radical cystectomy [7,8]. Selectively increasing bladder tumor sensitivity to radiation could improve the efficacy of this treatment, limiting its side effects and reducing the need for cystectomy. However, there is a limited number of experimental studies investigating radiosensitization strategies of bladder tumors, which highlights the urgent need to perform additional studies and optimize this approach [9].

The TAM receptor family of Tyrosine Kinase Receptors (RTKs) consists of TYRO3, AXL and MERTK. The TAM family has evolved the most recently from the 20 known classes of RTKs, and is distinct by a unique conserved sequence in their tyrosine kinase domain (TKD): KW(I/L) A (I/L) ES [10]. The physiological role of TAM receptors is to negatively regulate innate immune response and to perform the clearance of apoptotic cells or efferocytosis [11]. TYRO3 specifically is known to be abundantly expressed in the brain, lungs and bone marrow and to regulate such physiological processes as neuron protection from excitotoxic injury, platelet aggregation and cytoskeletal reorganization. In addition, TYRO3 plays a significant role in inhibition of Toll-like receptors (TLRs)-mediated innate immune response [12]. Cancer cells are shown to upregulate TAM receptors to escape the immune system attack, furthermore they play a significant role in promoting cancer cell growth, survival, and metastatic spread of several tumor types. In recent years several studies have established their involvement in the resolution of inflammation, autoimmunity and tumor immunology [13,14].

It has been shown that several TAM receptors can modulate radiation response [15]. For example, targeting AXL successfully improved radiation response in head and neck squamous cell carcinoma (HNSCC) [16], and it has also been found to be upregulated and activated in induced radioresistant HNSCC cell lines [17]. A tyrosine kinase AXL inhibitor R428 (BGB324) was found to reverse radiation resistance in glioblastoma cell lines [18]. A Phase I clinical trial using this AXL inhibitor is currently being tested in glioblastoma which progressed after radiation and other combined treatments (NCT03965494). UNC2025, a potent MERTK inhibitor, has shown efficacy in leukemia preclinical models alone and in combination with chemotherapy [19]. Regarding TYRO3, no specific inhibitor has been developed.

We have previously demonstrated that TYRO3 is overexpressed in 50% of BCa and that TYRO3 overexpression conferred a TYRO3-dependence to bladder tumor cells both in vitro and in vivo [20]. Another study has demonstrated that overexpression of GAS6, a potent TAM ligand stimulates proliferation and invasiveness of bladder cancer cells [21]. However, so far, no study has investigated the concurrent inhibition of TYRO3 or its ligand in radiation treatment.

In this study, we therefore sought to determine whether the modulation of TYRO3 expression could impact the radiation response of BCa. For this, we evaluated the influence of the expression levels of TYRO3 on colony or cell survival assays, DNA damage, γH2AX foci formation, gene expression profiling and cell cycle regulation after radiation on different BCa cell models.

## 2. Results

### 2.1. TYRO3 Modulation Influences Radiosensitivity of Bladder Cancer Cell Lines

We first evaluated the intrinsic radiosensitivity using colony forming or proliferation assay in a panel of BCa cell lines with different TYRO3 expression protein levels (Supplementary Figure S1). Cells were exposed to increasing doses of radiation and the intrinsic radiosensitivity among the cell lines were compared using the D_10_ values (dose to achieve 10% cell survival). The D_10_ of this panel of cell lines ranged between 4.7 Gy ± 0.43 and 7.2 Gy ± 0.33 (Supplementary Figure S1A). There was no correlation between TYRO3 expression (neither that of the other TAM receptor expression) and the intrinsic radiation response (Supplementary Figure S1A,B), so we thus selected three BCa cell lines, two TYRO3-high expressing and a TYRO3-low expressing UM-UC-3 cell line for further investigation of the impact of the modulation of TYRO3 expression on cell sensitivity to radiation.

**Figure 1:**
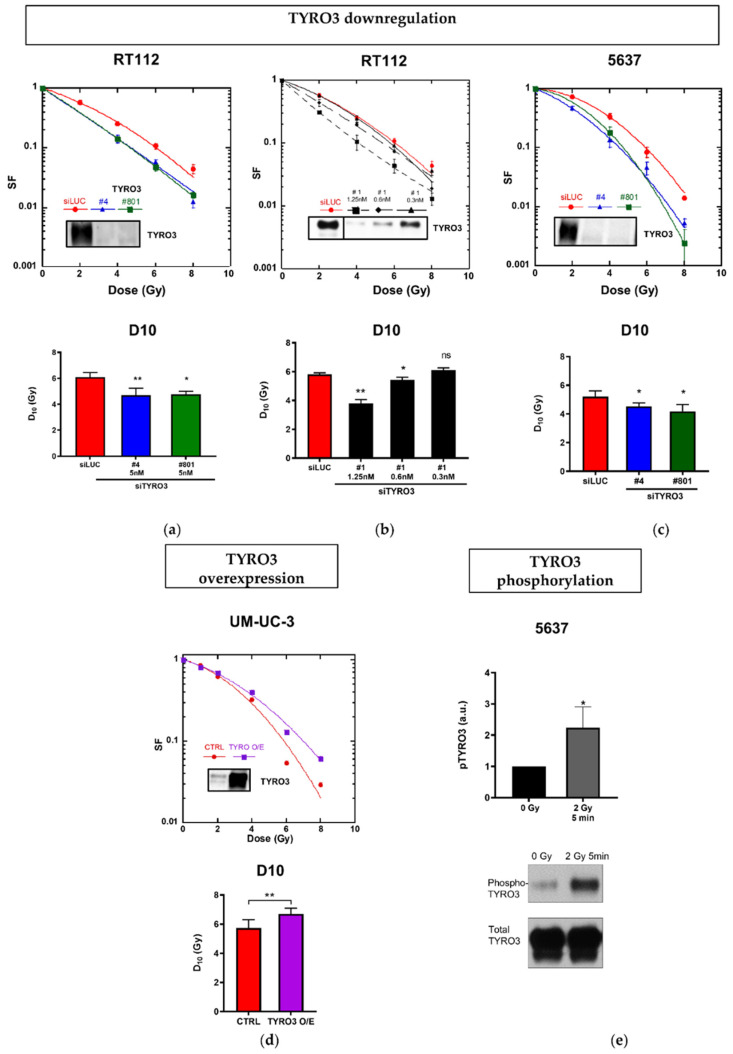
Impact of the modulation of TYRO3 expression on the radiosensitivity of bladder cancer cell lines. (**a**–**c**) Representative clonogenic survival curves of RT112 and 5637 cell lines (**upper** panel). 48 h after transfection with siLUC (control, red), siTYRO3#4 (blue), siTYRO3#801 (green) or diluted concentration of siRNA#1 (black) cells were exposed to increased doses of gamma-rays. In each case, downregulation was confirmed by western blot at 48 h after transfection. The corresponding D_10_ values were calculated after fitting the experimental data to the classical linear-quadratic equation (**lower** panel); (**d**) Representative survival curves of UM-UC-3 control (empty plasmid, red) and TYRO3 over-expressed cell lines (TYRO3 encoding- plasmid, purple) (**upper** panel). The upregulation was confirmed by western blot. The corresponding D_10_ values were calculated after fitting the experimental data to the classical linear-quadratic equation (**lower** panel); (**e**) Phosphorylated TYRO3 shown in western blot (**lower** panel) after immunoprecipitation of the cell extracts with phospho-Tyrosine antibodies and its relative expression in 5637 cells quantified using Image-J software and normalized to that of total TYRO3 (**upper** panel). Data represents mean ± SD of 3 independent experiments. Unpaired t-test analysis: * *p* < 0.05; ** *p* < 0.005, ns: not significant.

In the TYRO3-high expressing RT112 and 5637 cell lines, TYRO3 was downregulated using 3 different siRNAs targeting TYRO3 and with siLUC (non-specific siRNA) used as a control. 48 h after transfection, the expression of TYRO3 was efficiently downregulated (Figure 1a–c). The cells were then irradiated with increasing doses of radiation (2–8 Gy) and survival fractions assessed by clonogenic assay (Figure 1a–c). The D_10_ showed a statistically significant decrease when TYRO3 was drastically downregulated with 2 different siRNA (siTYRO3 #4 and #801) in both cell lines. We previously showed that siTYRO3#1 reduced BCa tumor growth in vivo and strongly impacted cell viability of RT112 and four other BCa cell lines [20]. We found that 5 nM of siTYRO3#1 did not allow sufficient cell survival (without irradiation) to assess the radiation response. However, when siTYRO3#1 was serially diluted on RT112 cell line and combined with radiation, it resulted in a dose dependent decrease in the D_10_ leading to a significant radiosensitization (Figure 1b). We verified that the two other TAM family members, AXL and MERTK levels were not impacted by siTYRO3 #4 and siTYRO3#801 (Supplementary Figure S2).

Conversely, when TYRO3 was over-expressed (O/E) in the TYRO3-low expressing cell line (UM-UC-3), the UM-UC-3-TYRO3 O/E cells were significantly more radioresistant than empty vector-transfected cells used as control (D_10_ = 6.6 Gy ± 0.4 vs. 5.5 Gy ± 0.4, from three independent experiments) (Figure 1d).

The activation of membrane receptor tyrosine kinases (RTKs) occurs usually within minutes after irradiation and belongs to the earliest events characterized next to the recognition of DNA damage (reviewed by [22]). We examined the activation of TYRO3 following ionising radiation in 5637 cells by western blot after immunoprecipitation of phospho-tyrosine complexes. We observed that after 2 Gy irradiation, TYRO3 phosphorylation levels were significantly increased within 5 min following irradiation (Figure 1e).

### 2.2. TYRO3 Modulation Impacts Long-Term Persistence of Ionising Radiation-Induced Foci That Is Not Correlated with an Accumulation of Unrepaired DNA Damage

**Figure 2:**
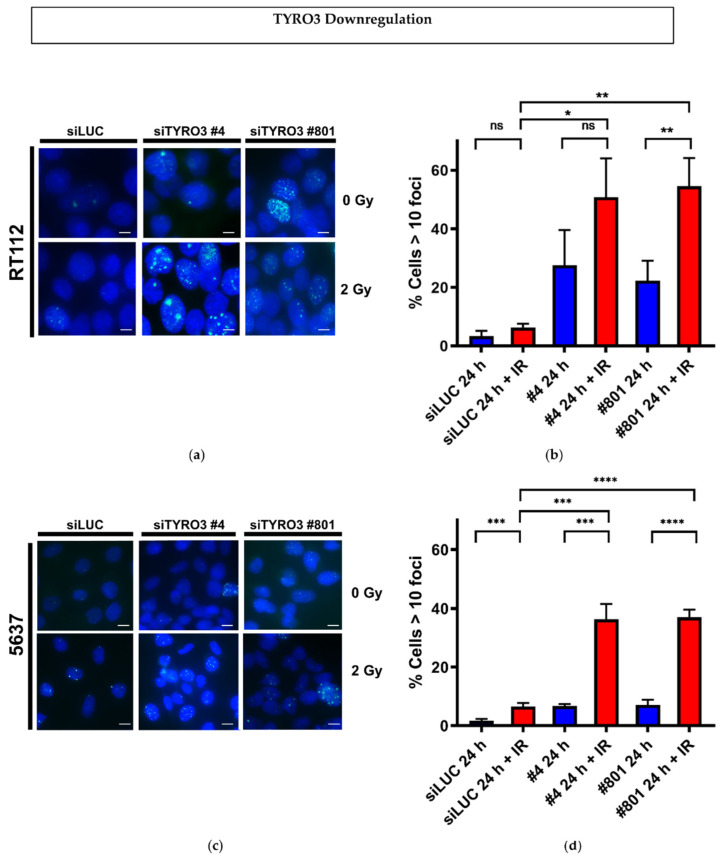

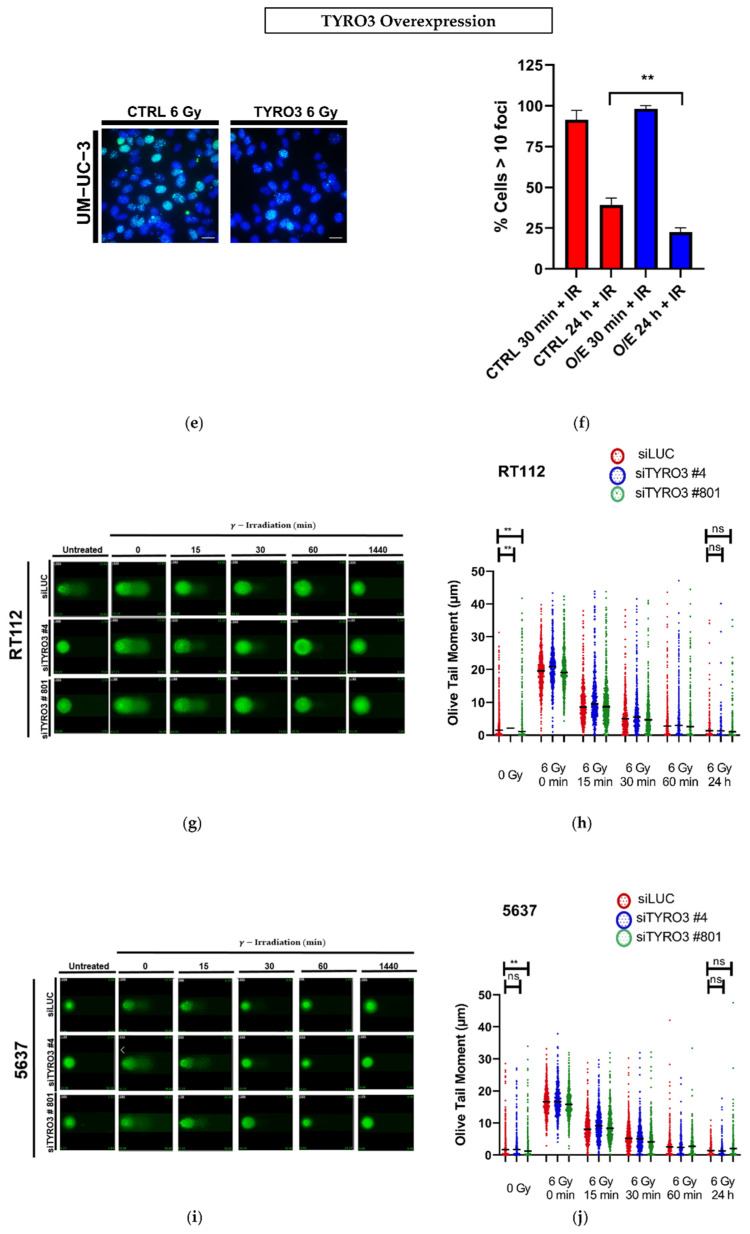

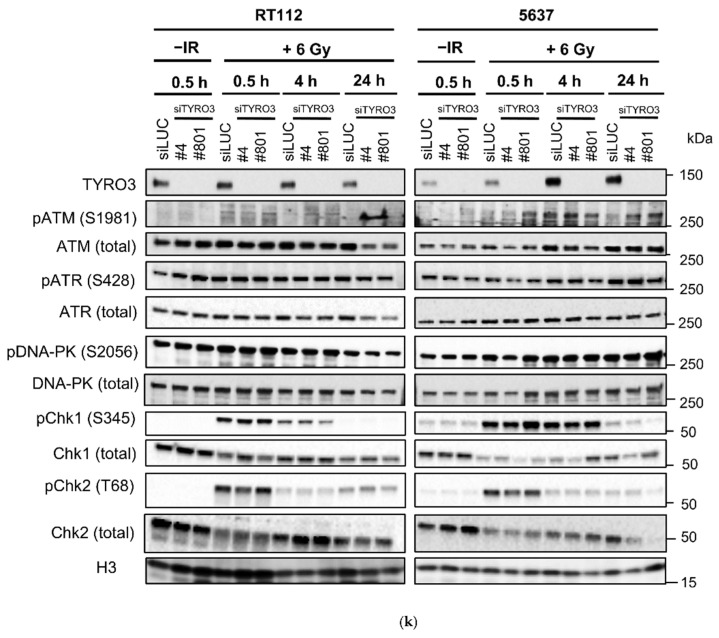
TYRO3 modulation and its impact on Ionizing Radiation-Induced Foci and DNA damage. γH2AX foci visualized after 24 h of 2 Gy irradiation in TYRO3 downregulated RT112 (**a**) or 5637 (**c**) cells (scale bar 5 microns); Quantification of cells containing more than 10 γH2AX foci at 24 h after 2 Gy of irradiation in the downregulated RT112 (**b**) and 5637 (**d**) cell lines; (**e**) γH2AX foci visualized after 24 h of 2 Gy irradiation in TYRO3 overexpressing UM-UC-3 cell line (Scale bar 5 microns); (**f**) Quantification of cells containing more than 10 γH2AX foci at 30 min and 24 h after 2 Gy of irradiation in TYRO3 overexpressing cell lines. The data shown above is from three different experiments and error bars represent the SD. Unpaired *t*-test analysis: * *p* < 0.05; ** *p* < 0.005; *** *p* < 0.0005; **** *p* < 0.0005, ns: non-significant. Representative images of the alkaline Comet assay performed on TYRO3 downregulated RT112 (**g**) and 5637 (**h**) cells and the resulting Olive tail moments analysis in TYRO3 downregulated RT112 (**i**) and 5637 (**j**) cells irradiated at 6 Gy. The data shown here is from three independent experiments analyzing 200 nucleus per condition per experiment, horizontal bars represent the median values. Kruskal-Wallis nonparametric tests with Multiple comparisons were used: * *p* < 0.05; ** *p* < 0.005, ns: non-significant; (**k**) western blot of DNA damage repair proteins; TYRO3 was downregulated, irradiated at 6 Gy and the lysates were prepared and analyzed 0.5–24 h after.

To decipher the mechanisms underlying the observed impact of the modulation of TYRO3 expression on the radiosensitivity of BCa, we studied the γH2AX foci formation and repair by immunofluorescence staining after TYRO3 upregulation in UM-UC-3 cells or downregulation in both RT112 and 5637 cells combined with radiation treatment. We analysed both foci at 30 min (where the foci formation is maximum) (Supplementary Figure S3) and 24 h after irradiation (residual foci) (Figure 2a–f). 30 min after irradiation, in both cell lines, TYRO3 downregulated cells induced an increase in these foci. We noticed also in RT112 and to a lesser extent in 5637 cells, that even in absence of radiation both siTYRO3 #4 and #801 induced γH2AX foci as soon as 48 h post-transfection (in blue, 30 min after IR in the Supplementary Figure S3). This effect was persistent during the following 24h (Figure 2a,c). We then analysed the residual foci 24 h after irradiation and observed an increased number of residual foci in both TYRO3 downregulated cell lines (Figure 2b,d). Indeed, between 36 to 37% of cells for 5637, and 50 to 54% of cells for RT112 still had more than 10 foci/nucleus after 24 h of 2 Gy irradiation in the combined treatment, whereas for the siLUC-transfected cells without radiation, almost all the induced DNA foci had been repaired 24 h after irradiation and only 5–9% of cells were still having more than 10 foci/nucleus (Figure 2b,d). Conversely, TYRO3 overexpression in UM-UC-3 cells did not impact the formation of initial foci but resulted in a significant decrease in number of residual foci when compared to the CTRL (23 vs. 39%, respectively) 24 h after 6 Gy radiation (Figure 2e,f).

In order to discriminate whether γH2AX foci were directly correlated with the extent of DNA lesions following TYRO3 downregulation and irradiation, we followed the DNA damage induction and repair using alkaline comet assay for RT112 and 5637 cell lines (Figure 2g–j). Immediately after irradiation (48 h after TYRO3 siRNAs transfection) irradiated transfected cells showed statistically significant differences in the distribution of the Olive tail moment in both RT112 and 5637 cell lines. However, this distribution decreased over time, and 24 h after irradiation, no difference was observed in siTYRO3 transfected cells as compared to control cells (Figure 2h,j) suggesting that TYRO3 depletion did not impair DNA repair. To validate these data at the protein level either in their expression or activation, we evaluated by western blot the key sensor proteins of DNA damage response ATM, ATR, DNA-PK, Chk1 and Chk2 in both cell lines after downregulation of TYRO3 with or without irradiation. We did not observe altered expression nor phosphorylation levels of these proteins confirming the Comet assay results (Figure 2k).

### 2.3. Comparative Gene Expression Analysis of RT112 and 5637 Irradiated Cells after TYRO3 Downregulation

**Figure 3:**
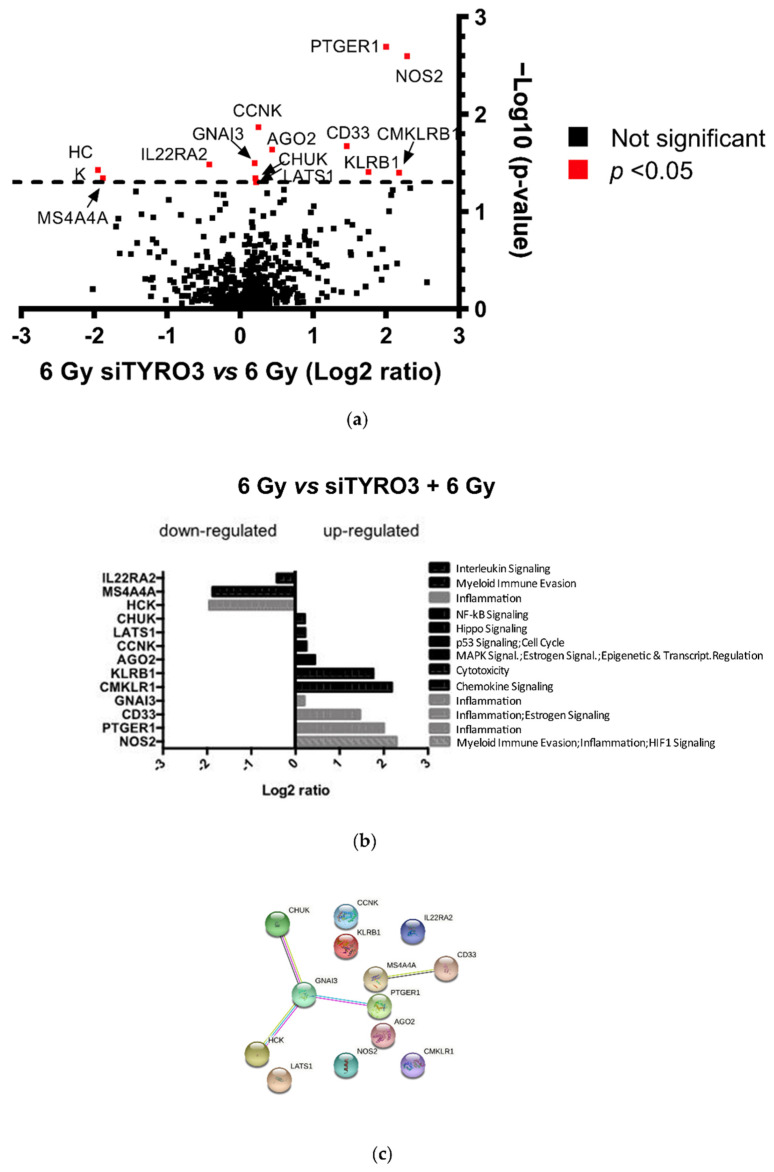
TYRO3 modulation and its impact on DNA damage response pathways. (**a**) Volcano plot showing the fold change (Log2 Ratio) versus negative log of the p-value of differentially expressed genes after Nanostring analysis between BCa (RT112 and 5637) 6 Gy irradiated cells versus BCa 6 Gy irradiated cells after complete TYRO3 knock-down (siTYRO3#4 and siTYRO3#801). Significant: *p*-value < 0.05 (**b**) Log2 ratio of the significantly expressed genes (*p* < 0.05) after Nanostring analysis between the two groups. The name of the up- or downregulated genes are listed on the left. On the right are the corresponding Nanostring gene annotations. (**c**) Protein-protein association network of the up and downregulated genes assessed using the STRING database.

To further support our observation and to investigate the molecular mechanisms underlying the radiosensitization observed after TYRO3 depletion, we used the Nanostring-based RNA assay to study gene expression levels. NanoString nCounter is a multiplex nucleic acid hybridization technology already used on clinical samples that enables reliable and reproducible assessment of the expression of up to 800 genes in a single assay [23]. Using the Tumor Signaling 360^TM^ panel containing 760 genes involved in 48 cancer pathways, we identified only 13 genes (10 upregulated and 3 downregulated) as differentially expressed (*p* value ≤ 0.05) in both cell lines following 6 Gy irradiation between non-transfected cells and transfected cells with the two siTYRO3 (Figure 3a,b—the complete table of the Log2 fold-change and p-value between the 2 groups is given in Supplementary Table S2). An analysis of this list of 13 genes with STRING protein-protein interaction network (https://string-db.org/, accessed on 8 April 2022) (Figure 3c and Supplementary Table S2) did not identify any modification regarding the DNA repair pathways present in the Tumor Signaling 360^TM^ panel, confirming previous results. TYRO3-regulated genes after irradiation involved mainly the “inflammation” gene set (5 genes over 10 upregulated) and none of the modified gene are known to directly impact the radiation response. The only significantly differentially expressed gene linked to the DNA damage response between the two groups is *CCNK*, which encodes for the Cyclin K, a member of the Cyclin family involved in cell cycle regulation.

### 2.4. TYRO3 Downregulation Affects Cell Cycle, Causes More Cell Accumulation in G2/M Phase Following Irradiation and Disrupts Mitosis Regulation

**Figure 4:**
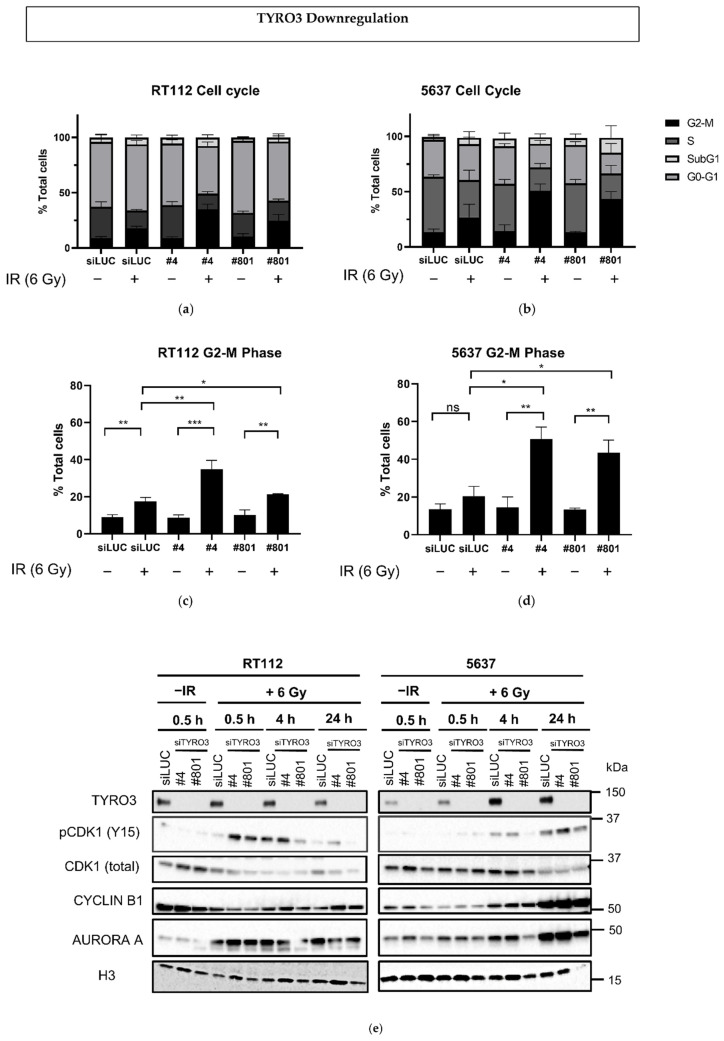
TYRO3 downregulation affects cell cycle following irradiation. Analysis of the cell cycle distribution 24 h after 6 Gy irradiation on TYRO3-downregulated RT112 (**a**) and 5637 (**b**) cells. Comparison of percentage (%) of cells in G2/M in RT112 (**c**) and 5637 (**d**) cells. Data shown is from three different experiments and error bars represent the SD. Unpaired t-test analysis: * *p* < 0.05; ** *p* < 0.005; *** *p* < 0.0005, ns: non-significant. (**e**) western blot of cell cycle proteins 30 min and up to 24 h after irradiation.

As a member of the cyclin family of proteins was identified as differently expressed gene, we decided to explore the cell cycle distribution after siTYRO3 downregulation and radiation. BCa cell lines were treated with siLUC or siTYRO3 for 48 h, then irradiated with 6 Gy or mock-irradiated and analyzed by flow cytometry 24 h after. At this time point, TYRO3 downregulation alone did not significantly affect the cell cycle distribution and cells remained largely in G0-G1 (55–65%) for RT112 (Figure 4a) and in S phase (42–50%) for 5637 (Figure 4b). Radiation alone induced moderate increase in percentage cells in G2/M phase in the siLUC transfected cells (by 8.6% and 7.0% in RT112 and 5637 cell lines, respectively) as expected in p53-mutated cell lines (Figure 4c,d). However, when TYRO3 downregulation by different siRNAs was coupled with radiation, it resulted in significant increase in cells in G2/M phase (by 11.0–26.3% for the RT112 and 30.1–36.2% for the 5637 depending on the siTYRO3 used (Figure 4c,d and Supplementary Tables S3 and S4).

The significant increase in the cells in the G2/M phase of the cell cycle in transfected cells 24 h after irradiation led us to evaluate key proteins involved in the G2/M transition and the mitotic spindle assembly. For this, the expression levels of AURORA A kinase, CDK1, phospho-CDK1 and CYCLIN B1 were examined (Figure 4e,f). The phosphorylation of CDK1 (Y15) was detected by 30 min and up to 24 h after irradiation depending on the cell line and the siRNA used. The expression levels of CYCLIN B1 and AURORA A increased as early as 30 min following irradiation (AURORA A) and depending on the cell line were growing over time to culminate 24 h after irradiation on both cell lines with both siRNA, leading to the conclusion that downregulation of TYRO3 might disrupt mitosis regulation through differential mitotic protein expression levels.

## 3. Discussion

To foster the use of the emerging bladder preserving therapies, there is growing interest to improve the current chemo-radiotherapy regiments by sensitizing BCa cells towards one of these modalities. The major aim of this study was to investigate the consequences of combining radiation therapy with TYRO3 downregulation in BCa.

TYRO3 belongs to the TAM (TYRO3, AXL and MERTK) family of RTKs and has been less frequently studied than the other two members of the TAM family. Recently, we demonstrated that TYRO3 is upregulated in 50% of MIBCs and that TYRO3 overexpression conferred a TYRO3-dependance to bladder tumor cells [20]. Despite the wealth of literature and date implicating the role of TYRO3 in cancers, there are no studies that have investigated TYRO3 therapeutic potential in combination with radiation treatment. A recent study reported that overexpression of GAS6, a potent TAM ligand stimulates proliferation and invasiveness of bladder cancer cells [21]. The authors found that GAS6 downregulated decreased proliferation of BCa cells, similar to the effects found after TYRO3 downregulation observed here and by Dufour and colleagues [20]. We also found that TYRO3 knockdown induced BCa cell cycle arrest. However, in contrast to Mao and colleagues’ work [21], TYRO3 signaling seems not to be strongly regulated via the PI3K-AKT signaling pathway as no significant positive correlation with the PI3K family gene expression were found in our gene expression analysis. Also, GAS6 knockdown was observed to induce BCa cell cycle in the G1 phase, while we observed significant arrest in the G2/M phase. Although different BCa cell lines were used in the respective studies, it might suggest that GAS6 and TYRO3 inhibition have different effects, which is plausible considering that GAS6 is also a potent receptor binding to AXL and MERTK [24,25] and that another TAM ligand, Protein S1 might be implicated [26]. Moreover, three additional ligands potent to bind TAM receptors have been discovered such as Galectin 3 (Gal3) [27], Tubby and tubby-like protein 1 (Tulp1) not investigated in these studies [28].

Receptor tyrosine kinases (RTKs) have been identified to play role in early cell response to radiation, inducing secondary activation of cytoplasmic protein kinase signalling cascades. This activation and their downstream effectors contribute to tumor cell resistance to treatments via either pro-proliferative or anti-apoptotic signals depending on the tumor type [17,24]. In addition, we found that as many other RTKs, TYRO3 is phosphorylated within minutes after irradiation. Downregulation of TYRO3 resulted in increased radiosensitivity of BCa cells, while overexpression resulted in increased radioresistance, demonstrating that TYRO3 plays a role in radiation response. Our results are the first report to identify TYRO3 as a mediator of radiation response in BCa. Similar observations have been made in Head and Neck Squamous Cell carcinoma (HNSCC) when AXL, another TAM receptor was downregulated or inhibited [16] leading to a radiosensitization. However, in this study, AXL was found to regulate the DNA repair pathways via AKT and DNA-PK activity. Our results lead to different conclusions as the combined treatment of TYRO3 downregulation and irradiation did not modify the activation or expression levels of five different key DNA repair proteins including DNA-PK.

Among the genes differentially expressed (and linked to the DNA damage response) between the irradiated BCa models and the same irradiated models after TYRO3 downregulation is the CCNK gene encoding for the Cyclin K. The Cyclin K is known to regulate the phosphorylation of the C-terminal domain of RNA polymerase II, leading to the regulation of its transcriptional activity [29,30] and it has been shown that this regulation is functionally linked to the DNA damage response and the maintenance of genome stability [31,32]. One early event after TYRO3 downregulation combined with radiation could be the transcriptional regulation and mRNA processing via RNA Pol II modulation however further experiments are needed to confirm this hypothesis.

Modulation of the cell cycle distribution is one of the mechanisms leading to cell radiosensitization [33,34]. In our study, downregulation of TYRO3 combined with radiation increased the initial DNA damage but did not affect the DNA repair. This leads us to the conclusion that the persistent foci observed 24 h after irradiation could not be due to remaining DNA lesions. As shown by the cell cycle experiments, the combination of TYRO3 downregulation and radiation resulted in a remarkable increase in the G2/M phase of the cell cycle. This increase is supported by an increase in CYCLIN B1 and AURORA A levels in the combined treatment. It has been shown that BRCA1 interacts with CYCLIN B1, and that this interaction is modulated by DNA damage and cell cycle phase [35]. This allows us to conclude that the cell cycle block leads to the persistent γH2AX foci observed 24 h after radiation. Even if the phosphorylation of the H2AX histone variant (γH2AX) is a well-established marker of DNA stand breaks induced by DNA damaging agents, induction of these foci is not always accompanied by an increase in double-strand breaks [36,37]. This was also confirmed in this study. As G2/M is the most radiosensitive cell cycle phase [38], this could explain the radiosensitization observed and could be advantageous in clinical setting where fractionated radiation dose delivery is employed.

Due to the high structural similarities of the three TAM receptors, it is challenging to develop inhibitors specific for a single TAM receptor and a specific TYRO3 inhibitor has not yet been developed. Recently, in non-small cell lung cancer cells, the AXL inhibitor BGB324 (Bencentinib) has been shown to induce DNA damage and replication stress indicated by ATR/CHK1 phosphorylation [39]. As such modifications could not be observed in our study, the TYRO3 downregulation and the AXL inhibition do not share the same regulating pathways. However, targeting multiple TAM receptors may be a promising strategy, as all three TAM receptors stimulate macrophage polarization towards the cancer-promoting M2 phenotype, and therefore targeting multiple TAM receptors may have a stronger anti-cancer effect [40,41].

TAM inhibition has already been shown to potentiate the benefit from PD-L1 therapy when investigated in immunocompetent mice [42]. In this study, pan-TAM inhibitor treatment resulted in increased tumor-infiltrating leukocytes, M1-polarized intratumoral macrophages, and activation of natural killer cells in model of mouse colon carcinoma (CT26) and colon adenocarcinoma (MC-38). Interestingly, the same study concluded that pan-TAM inhibitor treatment does not result in tumor growth delay in immunocompromised mice, highlighting the importance of immune cell activity in the treatment response [42]. Following these encouraging results in mouse models, it would be interesting to explore the possibility to use for example an anti-PD1 strategy (i.e., Pembrolizumab) with an anti-TAM inhibitor treatment in BCa and perform immunoprofiling of the infiltrated immune cells before and after the treatments.

## 4. Materials and Methods

### 4.1. Cell Lines

The human BCa-derived cell lines 5637, RT112, UM-UC-5, UM-UC-9, VM-CUB-1, were obtained from DSMZ (Heidelberg, Germany), and UM-UC-3 from ATCC. UM-UC-3, UM-UC-5, UM-UC-9, VM-CUB-1 cells were cultured in DMEM (Life Technologies, Villebon-Sur-Yvette, France), whereas RT112 and 5637 cells were cultured in RPMI (Life Technologies). Media were supplemented with 10% fetal bovine serum (FBS, ThermoFisher Scientific, Courtaboeuf, France). Cells were kept at 37 °C, under an atmosphere containing 5% CO_2_. The identity of the cell lines used was checked by analyzing genomic alterations on comparative genomic hybridization arrays (CGH arrays) and sequencing genes known to be mutated: RAS, TP53, FGFR3 and PIK3CA. The cells were routinely checked for mycoplasma contamination.

Control and TYRO3-overexpressing cell lines were established by transfecting UM-UC-3 cells with a pCMV6-Entry vector (PS100001–Origene, Rockville, MD, USA) or a pCMV6-Entry vector encoding for the human TYRO3 open reading frame (RC208260 –Origene, Rockville, MD, USA) using lipofectamine 2000 (11668–027–ThermoFischer Scientific, Courtaboeuf, France) following the manufacturer’s protocol (3:1 DNA:Lipofectamine 2000 ratio, 2 µg of DNA/well in 6-well plate). The selection of the transfected cell populations was performed using 1 mg/mL of Geneticin (10131035–ThermoFischer Scientific, Courtaboeuf, France).

### 4.2. siRNA Transfection

Cells in the exponential phase of growth were detached with accutase solution (Sigma-Aldrich, Saint-Quentin Fallavier, France) and between 0.5–1 × 10^6^ cells per T25 flask were reverse-transfected with 5 nM siRNA (or 0.6–5 nM for siTYRO3 #1) using Lipofectamine RNAi Max reagent (ThermoFisher Scientific, Courtaboeuf, France), in accordance with the manufacturer’s protocol. siRNAs targeting TYRO3 were purchased from ThermoFischer Scientific, Courtaboeuf, France, (siTYRO3 #1, siTYRO3 #801) and Qiagen (siTYRO3 #4). Then, the cells were seeded in 6-well plates (between 200–10,000 cells) for 48 h to achieve complete downregulation of TYRO3. For the control siRNA, we used a Qiagen control targeting luciferase (siLUC) (SI03650353- Qiagen, Courtaboeuf, France). 3 different siRNAs were chosen and conducted in parallel targeting different regions of TYRO3 sequence (encoding Ig-like (siTYRO3 #1), intramembrane region (siTYRO3 #4) or kinase domain (siTYRO3 #801). siRNA sequences are listed in Supplementary Table S1.

### 4.3. Clonogenic Cell Survival or Cell Proliferation Assay Following siRNA Transfection and Irradiation

48 h after transfection, cells were irradiated with γ rays from a ^137^Cs source (GSR-D1) at a dose rate of 1 Gy/min. After culturing for 7–10 days, the colonies were washed with PBS, fixed with 100% ethanol and stained with a Coomassie R-250 solution (0.5%). Colonies formed by at least 50 cells were counted. UM-UC-3 cell line was unable to form colonies. In this case, the control and the over-expressing cell lines were plated in 6-well plates in duplicate at the density of 2 × 10^4^ per well. They were left to adhere for 8 h, irradiated, and let to grow after irradiation for 5 days. Cells were then trypsinized and counted. Each condition was repeated on at least 3 separate experiments.

Surviving Fraction (SF) was calculated as the ratio of the cell/colony count relative to mock-treated cells. Survival curves were generated by fitting the SF to a linear-quadratic model: SF = exp (^−αD −βD2)^, where D is the dose, and α and β adjustable parameters characterizing the radiation response. For each experiment, the D_10_ (dose to achieve 10% cell survival) was calculated using these parameters. Calculations were performed by non-linear least-squares regression using KaleidaGraph software. For each experiment, the D_10_ (dose to achieve10% cell survival) was calculated using these parameters.

### 4.4. Comet Assay

Single cell gel electrophoresis (SCGE) was carried out by the Trevigen’s comet assay kit (4250–050–K) with slight modifications compared to the manufacturer’s protocol. Briefly, cell suspension (1.5 × 10^5^/mL) and molten LM agarose were prepared at 37 °C in 1:10 (*v*/*v*) ratio. From there, 50 μL was instantly poured onto the comet slide (4250–200–03) and kept at 4 °C in the dark for 10 min for agarose adhesion. Slides have been irradiated for 6 Gy (dose rate 1 Gy/min) with gamma ray (^137^Cs, GSR D1). The slides were placed in an ice-cold lysis solution for 60 min at 4 °C and then deeply immersed in freshly prepared alkaline unwinding solution (pH > 13: 200 mM NaOH, 1 mM EDTA) for 1 h at 4 °C in the dark. Subsequently, the slides were placed in an electrophoresis chamber filled with the alkaline solution, run at 28 V for 30 min, then immersed in a 70% ethanol solution for 5 min twice and dried for 10–15 min at +37 °C. SYBR green staining was performed to visualize the comets through transmission light microscope. Randomly, 200 comets per slide were visualized and analyzed by scoring various parameters such as head, tail and tail moment under light microscope with Comet Assay Metafer CometScan software (MetaSystem, Altlussheim, Germany). Olive tail moment was calculated as the product of the tail length and the fraction of total DNA in the tail for each comet and compared between conditions.

### 4.5. Immunoprecipitation

Cells were cultured in 10 cm dishes, irradiated at 2 Gy and lysed after 5 min using lysis buffer (50 mM Tris pH 7.5, 150 mM NaCl, 1% NP-40, 0.25% Na Deoxycholate, supplemented with complete proteases and phosphatases inhibitors (Roche, Boulogne-Billancourt, France )). Further, 2 mg of protein lysate was pre-cleared with 30 µL agarose protein G beads (1:2 diluted in lysis buffer) by incubating for 3 h at +4 °C on an orbital shaker. After, the cleared sample was transferred to a microcentrifuge tube containing 5 µg of IgG2b isotype control (MAB004—R&D Systems) or 2.5 µg anti-phosphotyrosine (03–7700- Invitrogen, ThermoFisher Scientific, Villebon-sur-Yvette, France) and 2.5 µg anti-phosphotyrosine (05–321-Merck—Millipore, Guyancourt, France) antibodies and left to incubate overnight at +4 °C on an orbital shaker. The following day, 30 µL agarose protein G beads (1:2 diluted in lysis buffer) were added to the sample and incubated for 1 h at +4 °C on an orbital shaker. After 5 washes with lysis buffer, the supernatant was aspirated and 25 µL of warm loading buffer was added to the beads, vortexed and boiled for 10 min. 15 µL of sample was loaded in SDS-PAGE gel and electrophoresed. The sample was transferred to a nitrocellulose membrane and probed with TYRO3 antibody.

### 4.6. Immunofluorescence Microscopy

The γH2AX foci formation was evaluated at 30 min or 24 h after irradiation of 2 or 6 Gy. The cells were fixed with 4% paraformaldehyde for 15 min, treated with 0.5% Triton X-100 for 15 min, blocked with 5% BSA for 1 h, and incubated with an anti-γH2AX antibody (Merck, dilution 1:1000) for 1 h. These cells were then labelled with Alexa Fluor antibody (Invitrogen, ThermoFisher Scientific, Villebon-sur-Yvette, France) for 45 min and counterstained with ProLong Gold Antifade Mounting medium with DAPI (Invitrogen, ThermoFisher Scientific, Villebon-sur-Yvette, France). Foci were visualized with a 3D-SIM immunofluorescence microscope and analyzed by ImageJ, using an optimized macro script. Over 300 cells were counted from at least three random fields photographed at the same exposure time under a × 60 objective for respective experiments, and the percentage of cells containing >10 fluorescent foci was calculated.

### 4.7. Flow Cytometry

Cell cycle was evaluated using dual staining by 5-bromo-2′-deoxyuridine (BrdU) and propidium iodide (PI). siRNA transfected treated cells were exposed to radiation or mock treatment and 24 h after were subjected to incubation with 10 µM BrdU for 30 min (#19–160, Merck—Millipore, Guyancourt, France). After, cells were harvested by trypsin treatment and fixed in 70% EtOH. After fixation, cells were washed and rehydrated with PBS (supplemented with 5% FBS) and subjected to pepsin digestion. Hydrolysis with 2N HCl was performed and continued with permeabilization by using 10 mM HEPES, 0.5% Tween-20 and 5% Goat serum. Cells were then stained with mouse anti-BrdU-fluorescein isothiocyanate (FITC) monoclonal antibody (BU20A,11–5071–42, Invitrogen/eBioscience). Cell cycle analysis was performed on a FACS Canto II cytometer (Beckton-Dickinson, Pont de Claix, France) and analyzed using the FlowJo software (FlowJo Inc., Ashland, OR, USA).

### 4.8. NanoString nCounter System Processing and Gene Expression Analysis

RT112 and 5637 cells were seeded in 6-well plates and were reverse-transfected with 5nM siRNA#4 or siRNA#801 using Lipofectamine RNAi Max reagent (ThermoFisher Scientific, Courtaboeuf, France). After 48 h to achieve complete down regulation of TYRO3, cells were irradiated at 6 Gy (^137^Cs, GSR D1). 4 h after irradiation the cells were detached and frozen in liquid nitrogen until processing. Total RNA was extracted (miRNeasy kit—Qiagen, Courtaboeuf, France) and 100 ng of the purified high-quality RNA was hybridized with the Tumor Signaling 360^TM^ panel (Nanostring Technologies, Seattle, WA, USA) at 65 °C overnight. This premade panel contains 760 genes involved in 48 cancer pathways (among them: cell adhesion; EMT; cytotoxicity; autophagy; glucose, glutamine and lipid metabolisms; epigenetic and transcriptional regulation; senescence; cell cycle, DNA damage repair, p53 signaling, apoptosis, HIF1 signaling, MAPK signaling, PI3K-Akt signaling; …) as well as housekeeping genes (HK) and negative and positive controls.

Further purification and binding of the hybridized probes to the optical cartridge was performed on the nCounter PrepStation, and finally, the cartridge was scanned on the nCounter Digital Analyzer. Raw counts from each gene were imported into the NanoString nSolver 4.0 software and normalized against background, positive controls and 16 different HK genes (DNAJC14, ERCC3, GUSB, MRPL19, NRDE2, OAZ1, POLR2A, PSMC4, PUM1, SDHA, SF3A1, TBC1D10B, TBP, TFRC, TMUB2, UBB). Log2 fold change (FC) were obtained on normalized data from Nanostring nCounter analysis. Transcriptomic analyses were compared between 6 Gy irradiated cells (RT112 and 5637) and 6 Gy irradiated siRNA TYRO3 (siRNA#4 and siRNA#801) transfected cells (RT112 and 5637). Only gene alterations with significant *p* value of ≤0.05 were chosen as changing between the two groups.

### 4.9. Western Blot

Cell extracts were prepared as described previously [43]. Briefly, lyophilized proteins were solubilized in 1X Laemmli sample buffer and boiled for 10 min. Protein concentrations were determined with the Bio-Rad Bradford Protein Assay Kit (Bio-Rad, Marnes-la-Coquette, France) before immunoblotting. The following antibodies were purchased from Cell Signaling Technologies (Ozyme, Montigny-le Bretonneux, France): AXL (8661), ATM (2873), phospho-ATM (Ser1981)–(4526), ATR (2790), phospho-ATR (Ser428)–(2853), Chk1 (2360), phospho-Chk1 (Ser345)–(2348), Chk2 (2662), phospho-Chk2 (Thr68)–(2197), DNA-PKc (4602), MERTK (4319), TYRO3 (5585). Antibody against phospho-DNA PKc (Ser2056) (ab18192) was purchased from Abcam (Paris, France).

### 4.10. Statistical Analysis

All functional experiments were carried out twice or three times, in triplicate. For the comparison of data between cell lines, two-tailed t-tests were used. The control siRNA (Luciferase GL2 siRNA, (AM4627, Ambion, Applied Biosystems, Paris, France) or DMSO was used as the reference group. Data are expressed as means ± SD, and differences with a *p* value < 0.05 were considered statistically significant. For Comet assays, Kruskal-Wallis nonparametric tests with multiple comparisons were used and medians compared as the data distribution of tail moments obtained were non-Gaussian [44]. All statistical analyses were performed with Prism8.0 (GraphPad Software, Inc., San Diego, CA, USA).

## 5. Conclusions

In summary, we report that TYRO3 downregulation in combination with radiation treatment is an effective treatment in BCa cells in vitro, resulting in the accumulation of cells in G2/M phase, and increased γH2AX foci in the combined treatment without involvement of DNA repair. We showed that TYRO3 overexpression results in higher radioresistance and has significantly less γH2AX foci in the nucleus following irradiation. This is the first study to implicate and demonstrate a prominent role of TYRO3 in radiation response in BCa, indicating a promising sensitization marker that could be clinically employed in future treatments.

## Supplementary Materials

The supporting information can be downloaded at: https://www.mdpi.com/article/10.3390/ijms23158671/s1.
