# Supplementary material for: Tyro3 Targeting as a Radiosensitizing Strategy in Bladder Cancer through Cell Cycle Dysregulation
Source: Int J Mol Sci. 2022 Aug 4;23(15):8671. doi: 10.3390/ijms23158671 (PMC9368768; doi:10.3390/ijms23158671)
Supplement: Supplementary file 1 [file ijms-23-08671-s001.zip › ijms-1816523-supplementary.pdf]

Supplementary Materials:

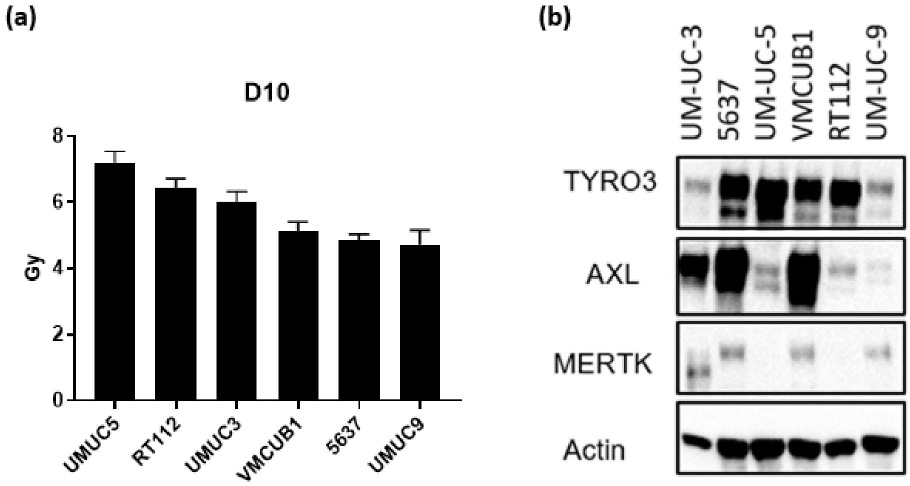

Figure S1. Radiosensitivity and TAM expression in BCa cell lines. D_{10} values of 6 BCa cell lines (**a**) and TAM receptor expression at protein level in BCa cell lines (**b**).

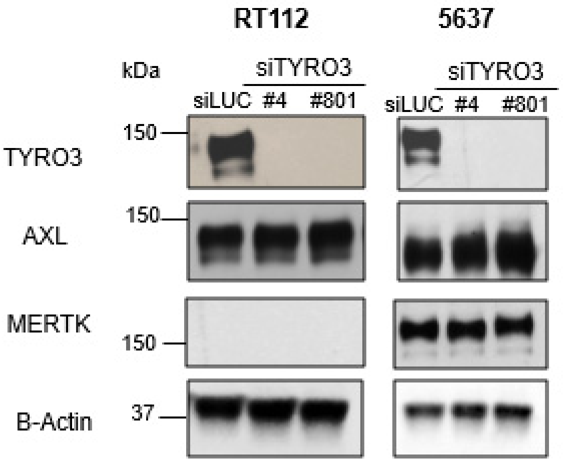

Figure S2. TAM expression levels following TYRO3 downregulation. siTYRO3#4 and siTYRO3#801 were used to downregulate TYRO3 and its impact on other TAM receptor expression was evaluated.

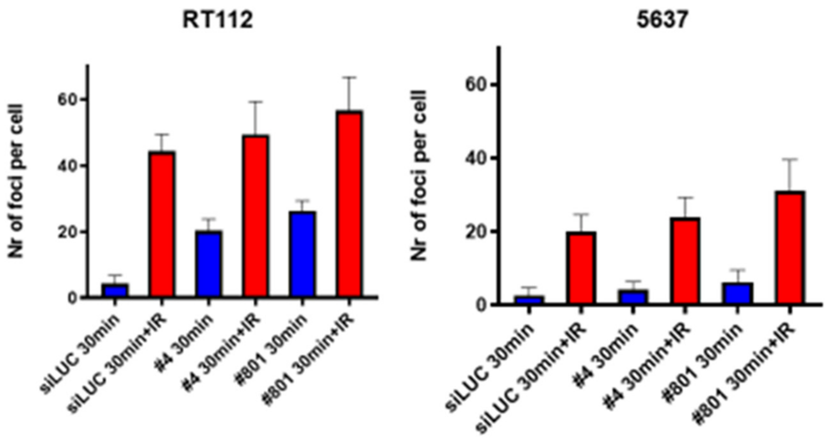

Figure S3. γH2AX foci 30 min after 2Gy or mock irradiation in RT112 and 5637 cell lines. A representative experiment is shown.

Table S1. Sequences of siRNAs used in the study.

siRNA	Exons targeted	Manufacturer	Reference	Sense strand sequence	Antisense strand sequence
siLUC (Luciferase GL2 siRNA)		Qiagen	ID: 1022070	5′-CGUACGCGGAAUACUUCGATT-3′	5′-UCGAAGUAUUCCGCGUACGTT-3′
TYRO3 siRNA #1	3/4	Thermo Fisher Scientific	AM51331 ID: 802	5′-GGUGUGCCAUUUUUCACAGTT-3′	5′-CUGUGAAAAAUGGCACACCTT-3′
TYRO3 siRNA #4	10	Qiagen	SI03096212	5′-GCUUCGAAAGAGACGGAAATT-3′	5′-UUUCCGUCUCUUUCGAAGCAG-3′
TYRO3 siRNA #801	19	Thermo Fisher Scientific	AM51331 - 801	5′-GGUCUGAAUCUUCACCAUCTT-3′	5′-GAUGGUGAAGAUUCAGACCTG-3′

Table S3. An overview of the percentage (%) cell in G2/M Phase for RT112 cell line.

	siLUC 0Gy	siLUC 6 Gy	siTYRO3 #4 0Gy	siTYRO3 #4 6Gy	siTYRO3 #801 0Gy	siTYRO3 #801 6Gy
**%**	8.940	17.57	8.750	35.00	10.22	21.44
**S.D**	1.422	2.136	1.527	4.610	2.771	0.2326

Table S4. An overview of the percentage (%) cells in G2/M Phase for 5637 cell line.

	siLUC 0Gy	siLUC 6 Gy	siTYRO3 #4 0Gy	siTYRO3 #4 6Gy	siTYRO3 #801 0Gy	siTYRO3 #801 6Gy
**%**	13.48	20.48	14.58	50.74	13.33	43.41
**S.D**	2.861	5.254	5.550	6.335	0.8656	6.761
